# Supplementary material for: SGAT: Shuffle and graph attention based Siamese networks for visual tracking
Source: PLoS One. 2022 Nov 23;17(11):e0277064. doi: 10.1371/journal.pone.0277064 (PMC9683595; doi:10.1371/journal.pone.0277064)
Supplement: Attachment — Submitted filename: AuthorsReply_Revised.pdf [file pone.0277064.s001.pdf]

Paper No.: No.: PONE-D-22-21523
Title: SGAT: Shuffle and Graph Attention based Siamese Networks for Visual Tracking
Authors: Jun Wang, Limin Zhang, Wenshuang Zhang, Yuanyun Wang*, Chengzhi Deng

Dear Editor:

We would like to thank the reviewers and you for your great efforts in helping us to improve the quality of the paper. After carefully considering the reviewers' comments and suggestions, we have significantly revised the paper with more details and descriptions.

A detailed summary of the revisions and some specific comments/responses are given in the following.

In short, we feel that we have addressed all crucial concerns of the reviewers. However，if you have any questions or further requirements, please do not hesitate to contact us.

Best regards,

Yuanyun Wang

October, 3, 2022

A Response and Summary of the Revisions: No.: PONE-D-22-21523

Authors' Responses:

****
Associate Editor
****

（1）Please ensure that your manuscript meets PLOS ONE's style requirements, including those for file naming. The PLOS ONE style templates can be found at https://journals.plos.org/plosone/s/file?id=wjVg/PLOSOne_formatting_sample_main_body.pdf and https://journals.plos.org/plosone/s/file?id=ba62/PLOSOne_formatting_sample_title_authors_affiliations.pdf

RESPONSE: Thanks. We have carefully read the guidelines and style templates. Then, we have modified the manuscript throughout the paper according to the templates.

(2) Please note that PLOS ONE has specific guidelines on code sharing for submissions in which author-generated code underpins the findings in the manuscript. In these cases, all author-generated code must be made available without restrictions upon publication of the work. Please review our guidelines at https://journals.plos.org/plosone/s/materials-and-software-sharing#loc-sharing-code and ensure that your code is shared in a way that follows best practice and facilitates reproducibility and reuse.

RESPONSE: Thanks for pointing this out. We will provide the appropriate code in three months.

****
Reviewer #1
****
(1) Abstract is very general. It has to be elaborated with characteristics of the results obtained.

RESPONSE: Thanks for the comments. We have re-organized the abstract in the revised manuscript with more details according to the results obtained.

...In this paper, we propose a novel tracking algorithm for feature extraction of target templates and search region images through convolutional neural networks and shuffle attention, and computes the similarity between the template and a search region through a graph attention matching. ...
…Extensive experiments demonstrate that the proposed tracking algorithm achieves

excellent tracking results on multiple challenging benchmarks. Compared with other state-of-the-art methods, the proposed tracking algorithm achieves excellent tracking performance. …

(2) No need to give specification for cl and reg in figure 1. It has to be explained through literature.

RESPONSE: Thanks for the valuable suggestion. We removed the branches cls and reg in the revised manuscript as shown in Fig 1. We have explained both the classification and regression branches in Section 2, i.e., related works. In addition, this prediction head is the usual way of mainstream trackers, including SiamRPN[12] and SiamCAR[16], etc.

SiamRPN has the classification (cl) and regression (reg) branches. The classification branch distinguishes the target from the surrounding background. And the regression branch refines the target location.

(3) In table 1 also expansion for GM, SA are not needed. Expansions need to be given during first refereed place.

RESPONSE: Thanks for the comments. We give the expansions of GM and SA during first refereed place.

In Table 1, we demonstrate the effectiveness of GM by ablation experiments. For more details, GM and SA are explained in Page 4 at the first refereed place, please see Section 3 and Section 4.2. In addition, we fixed the GM constant and validated the effectiveness of the SA module.
…2) the shuffle attention mechanism model (SA Unit), which reconstructs the basis features to focus on the target region and suppress the background interference through the spatial and channel-wise transformation; 3) graph attention matching (GM), which computes the similarity between the target template and a search region, and joints classification and regression branches to locate the target position in the current frame. …

(4) In table 1, what Success represents? What about accuracy?

RESPONSE: Thanks for pointing this out. In the evaluation metric, we explain the relevant definitions of success and accuracy. Among them, precision and accuracy denote the same concept. For more details, please see the Section 4.1.

…The precision is evaluated by the center location error (CLE) between the predicted location and the ground truth location. The precision plots are drawn in according to the frame percentages of CLE under the specified thresholds. Besides, the success rate is defined as the intersection over union (IoU) between the predicted bounding boxes

and the ground truth. Meanwhile, when the IoU exceeds a certain threshold, it is considered to track the target accurately, and the success plot is drawn by the frame percentage. …

(5) Figure 3, more explanation is needed like the reason for SGAT algorithm's best performance compared to all other methods.

RESPONSE: Thanks for the suggestions. We have added some sentences for analysis of superior performance in Fig 3, in the revised manuscript. For more details, please see the Section 4.3.

…since similarity learning based on graph matching effectively exploits the structured information, the SGAT algorithm achieves the best results in the success rate and different attributes. …

(6) Figure 4 is not clear.

RESPONSE: Thanks for pointing this out. We have drawn the figure again. And we have added some corresponding descriptions for Figure 4. For more details, please see the Fig 4 and Section 4.3.

…Here, the x-axis represents the 10th power of the tracking speed and the y-axis represents the success rate. For example, when x is taken as 2, the tracking speed is 200 frames per second. …

(7) Why the authors choose the threshold values 0.5 and 0.75. Justification is required.

RESPONSE: Thanks. In the paper associated with the GOT-10k dataset, the authors explicitly state the use of thresholds of 0.5 and 0.75, and subsequent mainstream trackers use this threshold for fair comparisons. The provision that all target trackers use the same training and testing sets provided by the dataset ensures a fair comparison of all trackers. GOT-10K training and testing sets are non-overlapping. After uploading the tracking results to the GOT-10K official website, the website automatically analyzes the tracking results. The assessment metrics provided include mean overlap rate (AO) and success rate (SR). SR0.5 indicates the rate of successful tracking frames with an overlap of more than 0.5, while SR0.75 indicates the rate of successful tracking frames with an overlap of more than 0.75. For more details, please see Section 4.3 in Page 9.

(8) The need for evaluation based on AO is required.

RESPONSE: Thanks for pointing this out.
We performed AO-based evaluation of the trackers on the GOT-10k dataset in Table

2 in Section 4.3. Please see the corresponding explain and analysis in Section 4.3

AO represents the average overlap between all estimated bounding boxes and ground-truth boxes. By using AO as an evaluation index, we can further evaluate the tracking performance of our tracker. Meantime, we used success rate for evaluation on OTB2015, LaSOT and UAV123 datasets, which is IoU-based method.

(9) What is the need for representing success rate as SR? Uniformity in representing evaluation measures are not followed.

RESPONSE: Thanks for the suggestions. We have revised all SR to success rate throughout the revised manuscript.

(10) Table 3 need to be elaborated. It is mentioned that UAV123 is used for evaluation. Why other state-of-the-art datasets are not used for evaluation?

RESPONSE: Thanks for pointing this out.

In UAV test set, the main challenge factors are occlusion and small targets, and most images have low resolution attributes. To the best of our knowledge, the state-of-the-art trackers are usually compared on the UAV dataset. Additionally, we also evaluate our tracker in OTB-100, GOT-10k and LaSOT. Extensive experimental results demonstrate that the proposed tracker has excellent performance on multiple benchmarks including OTB-100, GOT-10k, UAV123 and LaSOT, and outperforms many SOTA trackers. For more details, please see the Section 4.3.

(11) In some places, success is represented as AUC. Need to maintain uniformity.

RESPONSE: Thanks for suggestions. We revised the manuscript to maintain a uniform as success rate.

(12) Justification is required for fig 9.

RESPONSE: Thanks for suggestions.

Figure 9 shows the inability of our tracker to perform accurate localization in some complex scenarios, and we discuss it in more detail in Limitations.

... As shown in Fig 9, in complex tracking environment, trackers may occur tracking drift and tracking failure. In some extreme scenarios, the SGAT cannot complete the target tracking task well. For example, after the 106th frame in the sequence soccer and the 143rd frame in the sequence bird1, when there are many similar target interferences and long-term occlusion in the scene, the SGAT will lose the target and lead to tracking failure, which is also an urgent problem faced by the existing trackers.

Next, we will focus on the following two aspects: 1) modeling the target using spatial-temporal context information to ensure that the target can be located when occlusion occurs; 2) Adding a learnable memory unit to alleviate the problem that the target often disappears in long-term tracking. …

(13) Recent references need to be included.

RESPONSE: Thanks for comments. We added and discussed some key references in the revised manuscript, for example, Ref. [25], [26], [55], [56].

…In recent years, trackers based on Siamese network attract incremental attention for their leading performance [25-28]. …
…Fan et al. [55] propose a dual-margin model for accuracy and robust visual tracking, which formulated the target state prediction problem as a dual-margin model including an intra-object margin and an inter-object margin. Li et al. [56] propose a thermal infrared tracker based on a hierarchical spatially-aware twin network that regards the infrared tracking problem as a similarity verification task. …

[25] Hui, Le, et al. 3D Siamese Transformer Network for Single Object Tracking on Point Clouds. arXiv preprint arXiv:2207.11995. 2022.
[26] Tang F, Ling Q. Ranking-Based Siamese Visual Tracking. IEEE Conference on Computer Vision and Pattern Recognition. 2022: 8741-8750.
[55] Fan N, Li X, Zhou Z, Liu Q, He Z. Learning dual-margin model for visual tracking. Neural Networks. 2021: 344-354.
[56] Li X, Liu Q, Fan N, He Z, Wang H. Hierarchical Spatial-aware Siamese Network for Thermal Infrared Object Tracking. Knowledge-Based Systems. 2019: 71-81.

****
Reviewer #2
****

(1) In the abstract, the authors should introduce the core idea of the proposed method and point out the advantages of the method.

RESPONSE: Thanks for the comments.

We added more details in the abstract, including the core ideas of the paper and the advantages of the proposed approach.

...In this paper, we propose a novel tracking algorithm for feature extraction of target templates and search region images. Based on convolutional neural networks and shuffle attention, the tracking algorithm computes the similarity between the template and a search region through a graph attention matching. The proposed tracking algorithm exploits the correlations between the spatial and channel-wise information

to highlight the target region. Moreover, the graph matching can greatly alleviate the influences of appearance variations such as partial occlusions. …

(2) The motivation is not clearly in the introduction. What's the problem of this paper solved?

RESPONSE: Thanks for pointing this out. We further explain the motivation of design our tracking algorithm and describe the problem solved.

a. Most Siamese-based trackers use the features of the last convolution layer or cascaded multi-layers as the target representations of the template and the search region, which do not effectively use the structured and part-level information. To address this problem, we propose to combine the advantages of CNN and shuffle attention for feature representation of target templates and search region images.

b. Both the cross-correlation and depth cross-correlation take the template features as a whole for linear matching on the search regions, so that the adjacent sliding windows produce a similar response. To solve this problem, we introduced graph matching approach for similarity learning to mine more structured information.

Our tracking algorithm effectively uses the structured and part-level information and exploits structured and part-level information, which greatly alleviate the influences of appearance variations such as fast motion and partial occlusions.
For more details, please see the Section 1.

(3) Why the shuffle attention is better than the original channel and spatial attentions in tracking?

RESPONSE: Thanks.
The original spatial and channel attention does not take full advantage of the correlational attention between space and channel, making it less efficient, e.g., CBAM. The shuffle attention by dividing into different blocks along the channel is a lighter and more efficient way of integrating spatial and channel attention.

(4) There are several Siamese trackers and attentions should be discussed to enrich the related work, such as Learning dual-margin model for visual tracking, Learning Deep Multi-Level Similarity for Thermal Infrared Object Tracking, Hierarchical spatial-aware siamese network for thermal infrared object tracking, and learning dual-level deep representation for thermal infrared tracking.

RESPONSE: Thanks. We have updated the literature and further enriched the related work as suggested.

…In recent years, trackers based on Siamese network attract incremental attention for

their leading performance [25-28]. …

…Fan et al. [55] propose a dual-margin model for accuracy and robust visual tracking, which formulated the target state prediction problem as a dual-margin model including an intra-object margin and an inter-object margin. Li et al. [56] propose a thermal infrared tracker based on a hierarchical spatially-aware twin network that regards the infrared tracking problem as a similarity verification task. …

[27] Fan N, Li X, Zhou Z, et al. Learning dual-margin model for visual tracking. Neural Networks. 2021: 344-354.
[28] Liu Q, Li X, He Z, et al. Learning deep multi-level similarity for thermal infrared object tracking. IEEE Transactions on Multimedia. 2020: 2114-2126.
[55] Fan N, Li X, Zhou Z, Liu Q, He Z. Learning dual-margin model for visual tracking. Neural Networks. 2021: 344-354.
[56] Li X, Liu Q, Fan N, He Z, Wang H. Hierarchical Spatial-aware Siamese Network for Thermal Infrared Object Tracking. Knowledge-Based Systems. 2019: 71-81.

(5) What's the GM in Fig 1. There are missing the introduction about this.

RESPONSE: Thanks for pointing this out. We describe the details of the GM in section 3.3.

…we learn a graph attention matching (GM) based similarity measuring instead of cross-correlation. By decomposing the target template and search region features into multiple grids, and then computing the similarity of different template and search region grids, which greatly alleviate the challenging of pose variations of target. …

... we assume 1 × 1 × C grid of the feature map as a node. For node i on the template and node j in the search region, the correlation scores are. …

(6) How to divide the group in the shuffle attention module? The authors should explain the reason and conduct an ablation study.

RESPONSE: Thanks for the comments. We have added some sentences to descript how to divide the group in the shuffle attention module.

…As shown in Figure 2, the designed deep model effectively exploit the correlations between the spatial and channel-wise to highlight the target region without extra overhead. …

…It is assumed that there is a basis feature $r \in R^{C \times H \times W}$ obtained by convolutional neural network, where C, H and W represent the channel number, height and width, respectively. In the shuffle attention unit, a basic feature r is divided into D groups along the channel dimension, denoted as $r_k \in R^{C/D \times H \times W}$, $k \in \{1, 2, ..., D\}$,

where r_k represents the k sub-feature. In this way, the feature r is divided into multiple sub-features r_k, and then the weight coefficient of each sub-feature is learned through off-line training. Meanwhile, each sub-feature is internally divided into two branches, a and b, $r_k = [r_{ka}, r_{kb}]$, which uses the information correlations of spatial and channel-wise to learn the weight coefficient and reduce the redundancy of local features. …

…Channel transformation focuses on 'what' is important in an input image. The typical channel attention is SE block, which can effectively capture the correlation between channels. However, SE blocks usually increase the number of parameters of the model, which is not accord with the principle of lightweight design in tracking tasks. To generate channel weights efficiently, the spatial dimension of an input feature map is usually compressed, and adopt average-pooling to integrate spatial information. Based on prior information, we adopt a novel channel transformation method that resizes the channel-wise block through global average pooling. The channel-wise block is obtained as follows. …

…As a supplement to channel-wise transformation, spatial transformation aims to locate 'where' is an important region. To effectively carry out spatial transformation, the max-pooling and average-pooling are usually used to deal with input feature along channel dimension. In this paper, the specific implementation steps are as follows: firstly, group normalization (GN) is used to preprocess the spatial features. Then, linear transformation and activation function are combined to enhance the ability of feature representation and suppress the interference of background region. The transformed spatial features are as follows. …

…In shuffle attention module, we divide the basis features into multiple sub-features along the channel dimensions. The shuffle unit reconstructs each sub-feature by spatial and channel-wise transformations. Finally, the sub-features are combined by using the dependence along channel dimensions. …For more details, please see the Section 3.2 and 4.2.
