# Supplementary material for: SGAT: Shuffle and graph attention based Siamese networks for visual tracking
Source: PLoS One. 2022 Nov 23;17(11):e0277064. doi: 10.1371/journal.pone.0277064 (PMC9683595; doi:10.1371/journal.pone.0277064)
Supplement: Attachment — Submitted filename: AuthorsReply_Revised.pdf [file pone.0277064.s002.pdf]

Paper No.: **No.: PONE-D-22-21523R1**

Title: SGAT: Shuffle and Graph Attention based Siamese Networks for Visual Tracking

Authors: Jun Wang, Limin Zhang, Wenshuang Zhang, Yuanyun Wang, Chengzhi Deng

Dear Editor:

We would like to thank the reviewers and you for your great efforts in helping us to improve the quality of the paper. After carefully considering the reviewers' comments and suggestions, we have revised the paper with some details and references.

A detailed summary of the revisions and some specific comments/responses are given in the following.

In short, we feel that we have addressed all crucial concerns of the reviewers. However， if you have any questions or further requirements, please do not hesitate to contact us.

Best regards,

Yuanyun Wang

October,13, 2022

Response and Summary of the Revisions: No.: PONE-D-22-21523R1

****

Associate Editor

****

(1) Please include a reference to your own previously published work, https://journals.plos.org/plosone/article?id=10.1371/journal.pone.0273690 and clarify the motivations for this manuscript, in light of this previous work.

RESPONSE: Thanks. We included the precious work [22] in Reference.

[22] Yuanyun W, Wenshuang Z, Limin Z, Jun W. Siamese network with a depthwise over-parameterized convolutional layer for visual tracking. PLOS ONE. 2022;1-21.

And, we added some sentences to clarity the motivation in this manuscript and the difference between this and the previous work.

Different from the previous work [22], we design a novel feature extraction network based on GoogleNet to exploit correlations of the spatial and channel-wise information. Additionally, in order to alleviate the influences of appearance variations, we use a different similarity computing to obtain more accurate score maps. Inspired by above-mentioned works, in this paper, we propose a novel tracking algorithm based on shuffle attention mechanism and graph matching in Siamese network. The

shuffle attention mechanism in the backbone network reconstructs the basic features extracted from CNN, and makes the feature representation focusing on the regions of interest through spatial and channel-wise transformations. Different from the cross-correlation based similarity learning, the part-to-part graph attention matching further improves the tracking robustness in complex scenes, such as occlusion.

****
Reviewer #1
****

(1) Authors addressed all the comments specified by the reviewer. So, the paper can be accepted at this stage.

RESPONSE: Thanks for your comment.

****
Reviewer #2
****

(1) There are two same references [27] and [55] and there is missing a related reference 'Learning dual-level deep representation for thermal infrared tracking'.

RESPONSE: Thanks for pointing this out. We removed the same reference, and have included the key reference [3] in Introduction, Page 1. Some details are as follows:

[3] Liu Q, Yuan D, Fan N, et al. Learning dual-level deep representation for thermal infrared tracking. IEEE Transactions on Multimedia. 2022;1-8.

Visual tracking [1-3] is a fundamental research topic in computer vision. It aims to estimate target states in subsequent frames by given the initial state in the first frame. It is widely used in various applications, such as video surveillance [4], human-computer interaction [5], augmented reality [6], and so on. Recently, Convolutional Neural Network (CNN) is successfully used in visual tracking. Deep trackers [7,8] achieve robust tracking performance and real-time tracking speed. However, due to complicated appearance variations, visual tracking is still a challenging task.
